# Genome-wide association and selective sweep analyses reveal genetic loci for FCR of egg production traits in ducks

**DOI:** 10.1186/s12711-021-00684-5

**Published:** 2021-12-20

**Authors:** Hehe Liu, Lei Wang, Zhanbao Guo, Qian Xu, Wenlei Fan, Yaxi Xu, Jian Hu, Yunsheng Zhang, Jing Tang, Ming Xie, Zhengkui Zhou, Shuisheng Hou

**Affiliations:** 1 State Key Laboratory of Animal Nutrition; Key Laboratory of Animal (Poultry) Genetics Breeding and Reproduction, Ministry of Agriculture and Rural Affairs, Institute of Animal Science, Chinese Academy of Agricultural Sciences, Beijing, 100193 China; 2 Farm Animal Genetic Resources Exploration and Innovation Key Laboratory of Sichuan Province, College of Animal Science and Technology, Sichuan Agricultural University, Chengdu, 613000 China

## Abstract

**Background:**

As a major economic trait in poultry, egg production efficiency attracts widespread interest in breeding and production. However, limited information is available about the underlying genetic architecture of egg production traits in ducks. In this paper, we analyzed six egg production-related traits in 352 F_2_ ducks derived from reciprocal crosses between mallard and Pekin ducks.

**Results:**

Feed conversation ratio (FCR) was positively correlated with feed intake but negatively correlated with egg-related traits, including egg weight and egg production, both phenotypically and genetically. Estimates of pedigree-based heritability were higher than 0.2 for all traits investigated, except hip-width. Based on whole-genome sequencing data, we conducted genome-wide association studies to identify genomic regions associated with these traits. In total, 11 genomic regions were associated with FCR. No genomic regions were identified as significantly associated with hip-width, total feed intake, average daily feed intake, and total egg production. Analysis of selective sweeps between mallard and Pekin ducks confirmed three of these genomic regions on chromosomes 13, 3 and 6. Within these three regions, variants in candidate genes that were in linkage disequilibrium with the GWAS leader single nucleotide polymorphisms (SNPs) (Chr13:2,196,728, *P* = 7.05 × 10^–14^; Chr3:76,991,524, *P* = 1.06 × 10^–12^; Chr6:20,356,803, *P* = 1.14 × 10^–10^) were detected. Thus, we identified 31 potential candidate genes associated with FCR, among which the strongest candidates are those that are highly expressed in tissues involved in reproduction and nervous system functions of ducks: *CNTN4*, *CRBR*, *GPR63*, *KLHL32*, *FHL5*, *TRNT1*, *MANEA*, *NDUFAF4*, and *SCD*.

**Conclusions:**

For the first time, we report the identification of genomic regions that are associated with FCR in ducks and our results illustrate the genomic changes that occurred during their domestication and are involved in egg production efficiency.

**Supplementary Information:**

The online version contains supplementary material available at 10.1186/s12711-021-00684-5.

## Background

Eggs are considered an excellent source of animal protein, supplying proteins, fatty acids, vitamins, minerals, etc., in the daily lives of humans [1]. Thus, egg production is an important trait in poultry breeding and is favorably associated with reproduction efficiency and feed efficiency in egg-layers. Egg production traits include egg production amount, egg weight, feed/egg conversion ratio [2], and others. Some studies have identified quantitative trait loci (QTL) for these egg production traits [3–5]. In addition to genetic factors, several environmental factors, including nutrient level [6], lighting programs [7], and feeding management [8], have significant impacts on the egg production of poultry. In the past few decades, traditional selection based on phenotypic information has greatly improved poultry egg production but it has some limitations, including the fact that egg production traits can only be recorded in adult females and, therefore, these records arrive late for use in selection. In addition, the focus on cumulative egg production over longer time periods further delays the availability of phenotypes. Thus, achieving breeding progress in egg production efficiency through conventional selection using phenotypic information only is difficult.

In recent years, with the advances in molecular genetic technologies and the availability of DNA markers, identifying QTL that control egg production traits in poultry for application in marker-assisted selection (MAS) has progressed rapidly [9]. Combined with advances in nucleotide sequencing technologies and their decreasing costs, technologies such as chip array-based genotyping [10], reduced-representation genome sequencing [11, 12], and whole-genome sequencing can yield tens of thousands, hundreds of thousands, and even millions of genome-wide single nucleotide polymorphisms (SNPs), which help to unravel the genetic basis of individual differences in egg production efficiency. Depending on the availability of high-resolution SNPs, genome-wide association study (GWAS) is one of the most effective ways to identify important SNPs and candidate genes [13]. Based on GWAS, QTL for egg production traits in poultry can be screened much more quickly than before. Based on the records available in the chicken QTL database, to date, 600 QTL related to egg production traits have been identified [14].

Ducks (*Anas platyrynchos*) are one of the most important farm animals in China, efficiently providing meat and eggs for humans. All domesticated duck breeds were derived from the mallard duck in central China within a very short period, since about 500 B.C. [15]. The mallard duck has a small body size, low egg production rate, and high disease resistance. Under strong artificial selection, divergence between the mallard duck and the domesticated ducks has reached a high level for many traits, including body size, physiology, metabolic state [16–18], and, especially, egg production efficiency [19]. Pekin ducks with a big body size and high egg production efficiency are a commercial duck breed (including the Cherry Valley and Maple Leaf strains) that has diverged from the domestic duck through artificial selection and has become a world-famous breed with a high production performance in meat and eggs.

Although QTL for egg production traits have been extensively studied in poultry [14], the genetic basis underlying egg production traits has been little investigated in ducks. Moreover, the genomic changes that have led to the tremendous improvement in egg production efficiency of the Pekin duck during their domestication from the mallard duck are still largely unknown.

An F_2_ cross design can generate large genetic variation through recombination and has achieved great success in QTL mapping studies and GWAS for target traits, including in chickens [20, 21]. Therefore, the present study used an F_2_ duck population that was previously constructed by reciprocal crosses between mallards and Pekin ducks [18] to identify QTL for egg production traits in ducks and the genomic changes that occurred during domestication of the Pekin duck from the mallard by combining a GWAS and selective sweep analysis.

## Methods

### Ethics statements

All procedures for the experimentation and care of ducks were approved by the Animal Care and Use Committee of the Institute of Animal Sciences, Chinese Academy of Agricultural Science (CAAS). The methods and protocols were in accordance with their guidelines. All efforts were made to minimize suffering.

### Population and measurement of traits

Ducks that were previously produced by reciprocal crosses of mallards and Pekin ducks [18] were mated to produce 400 F_2_ ducks that were raised at the Pekin Duck Breeding Center of the Chinese Academy of Agricultural Sciences. In the orthogonal cross, 100 female mallards and 10 male Pekin ducks were selected as parents. In the reciprocal cross, four male mallards and 40 female Pekin ducks were selected as parents. All experimental ducks were raised under identical environmental and management conditions. Feed (pellet form) and water were provided *ad libitum*. The feed nutrient components are provided in Additional file 1: Table S1. During the laying period, lighting lasted 17 h per day, using artificial light as a complement, at a light intensity of 5 lx.

We recorded the total egg production (EP) of each female duck during their laying period that ranged from 226 to 329 days of age by using individual cages (see Additional file 2: Fig. S1). Between 230 and 267 days of age, eggs were collected every day, the cage number was marked on the eggshell, and eggs were individually weighed. The total egg weight (EW) for each female duck was the sum of the weights of the eggs produced during the 37 days. The difference subtraction method was used to calculate total feed intake (FI) during the test period, i.e., feed was provided to each individual cage by a special trough (see Additional file 2: Fig. S1) on the first day of the testing period (230-day-old), the remaining feed was weighed at the end of the testing period (267-day-old), and then subtracted from the initial weight to obtain the total feed intake for each duck over the test period. Average daily feed intake (DFI) was obtained by dividing FI by 37 days. Feed conversion ratio (FCR) was calculated as the ratio of total feed intake (FI) to total egg weight during the test period. Hip-width (HP) was measured on 35-week old ducks, when they were in the peak period of laying, as the straight length between the two ischial tubercles on both sides of the body, using a vernier caliper.

### Estimation of genetic parameters

We estimated genetic parameters for the F_2_ duck population using the multiple-trait derivative-free restricted maximum likelihood (MTDFREML) software and the following animal model [22]:

$$\mathbf{y}=\mathbf{X}\mathbf{b}+\mathbf{Z}\mathbf{a}+\mathbf{e},$$

where $\mathbf{y}$ is the vector of observations, $\mathbf{b}$ is the vector of fixed effects, $\mathbf{a}$ is the vector of genetic effects, $\mathbf{e}$ is the vector of random errors, and $\mathbf{X}$ and $\mathbf{Z}$ are the incidence matrices of fixed and genetic effects, respectively. In this analysis, batch was the fixed effect since the environment can vary between batches because of different feeding conditions:

$$\mathrm{Var}\left(\mathrm{a}\right)=\mathbf{A}{\upsigma }_{\mathrm{a}}^{2},$$

$$\mathrm{Var}\left(\mathbf{e}\right)=\mathbf{I}{\upsigma }_{\mathrm{e}}^{2},$$

where $\mathbf{A}$ is the additive relationship matrix that was constructed from the ducks’ pedigree data (recorded for three generations, including F_0_, F_1_ and F_2_), $\mathbf{I}$ is an identity matrix, and ${\upsigma }_{\mathrm{a}}^{2}$ and ${\upsigma }_{\mathrm{e}}^{2}$ are the direct additive genetic variance and the residual variance for the trait, respectively. Heritability and genetic correlations were estimated by using the following equations:

$${\mathrm{h}}^{2}=\frac{{\upsigma }_{\mathrm{\alpha }}^{2}}{\left({\upsigma }_{\mathrm{\alpha }}^{2}+{\upsigma }_{\mathrm{e}}^{2}\right)},$$

$${\mathrm{r}}_{\mathrm{a}}=\frac{{\upsigma }_{\mathrm{\alpha }1\mathrm{\alpha }2}}{{\upsigma }_{\mathrm{\alpha }1}^{2} {\upsigma }_{\mathrm{\alpha }2}^{2}},$$

where ${\upsigma }_{\mathrm{\alpha }1}^{2}$ and ${\upsigma }_{\mathrm{\alpha }2}^{2}$ are the additive genetic variances for traits 1 and 2, and ${\upsigma }_{\mathrm{\alpha }1\mathrm{\alpha }2}$ is the genetic covariance between the traits.

### Genomic sequencing, alignment, and variant calling

Blood was obtained from the wing veins of 352 F_2_ ducks and was rapidly frozen at − 20 °C. Genomic DNA was extracted using the standard phenol–chloroform protocol. The quality and quantity of DNA were assessed by using a Nanodrop spectrophotometer and agarose gel electrophoresis. For each sample, two paired-end libraries were generated using standard procedures according to the manufacturer’s protocols (Illumina, USA). The average insert size was 500 bp and the length of the reads was 150 bp. All libraries were sequenced on an Illumina^®^Hiseq X-Ten platform in a Bio-company (Berry Genomics, Pekin, China) to generate a raw read sequence coverage of 5×. These sequence data are stored in the Genome Sequence Archive (GSA) under the Project name of PRJCA003535 and have been published [23].

Adapter sequences and low-quality raw reads were removed by using the Trimmomatic (v0.36) software [24] with the following parameters: LEADING:20, TRAILING:20, SLIDINGWINDOW:4:20, and MINLEN:50. The resulting high-quality reads were mapped to the duck reference genome (IASCAAS_Peking Duck_PBH1.5, GCF_003850225.1) using the 'mem' algorithm of the Burrows–Wheeler Alignment (v0.7.12) tool with default parameters [25]. After mapping, SNPs and InDels were called using the GATK (version 3.5.0) HaplotypeCaller tool [26] with the following cut-off values: QUAL < 100.0, QD < 2.0, MQ < 40.0, FS > 60.0, SOR > 3.0, MQRankSum < − 12.5, and ReadPosRankSum < − 8.0. The output was further filtered using VCFtools (version 0.1.15) [27] based on the following criteria: (1) only SNPs with a minor allele frequency higher than 0.05 and a maximum allele frequency lower than 0.99 were retained; (2) the maximum missing rate was set at < 0.1; and (3) SNPs had to have only two alleles. After filtering, 9,584,532 SNPs remained and were distributed along the 29 autosomes, the Z and W sex chromosomes, and in Un (unplaced scaffolds), with a mean density of 8.5 SNPs/kb across the genome.

### Genome-wide association analysis

GWAS was performed on the F_2_ population to detect genomic regions that affect egg production traits in ducks, using the mixed linear model program EMMAX [28]. The linear model used to test each SNP individually was:

$$\mathbf{y}=\mathbf{X}{\varvec{\upalpha}}+\mathbf{Z}\upbeta +\mathbf{W}{\varvec{\upmu}}+\mathbf{e},$$

where $\mathbf{y}$ is the vector of observed phenotypes; $\mathbf{X}{\varvec{\upalpha}}$ represents the fixed effects, including the first three principal component values (PCA eigenvectors) derived from the whole-genome SNP genotypes, to correct for population stratification [28], and the batch effect; $\mathbf{Z}\upbeta$ represents the effect of the tested SNP, with $\upbeta$ the allele substitution effect; $\mathbf{W}{\varvec{\upmu}}$ represents the random animal effect, with variance–covariance structure based on the kinship matrix estimated using the whole-genome SNP genotypes; and $\mathbf{e}$ is the vector of random residual errors. SNPs with a P-value that reached a *Bonferroni*-corrected threshold (− log_10_ (*P*) ≥ 8.16) were considered significant.

### Screening for signatures of selection

Whole-genome sequencing data of 96 ducks (62 Mallard and 34 Pekin ducks) were downloaded from NCBI (https://www.ncbi.nlm.nih.gov) (see Additional file 3: Table S2) [17, 18]. Variant mapping and calling steps were performed as described above.

To analyze the regions that have been affected by long-term selection and are associated with the domestication of the Pekin duck from the mallard, we used VCFtools v0.1.13 to calculate the fixation index (*F*_ST_) and the population nucleotide diversity ratio Pi (mallards/Pekin ducks). The average *F*_ST_ and Pi were calculated in 20-kb sliding windows with a 10-kb step. The logarithmic function was used to transform the Pi ratio. We considered the windows with the top 5% values for the *F*_ST_ and log_2_ (θπ ratio) simultaneously as candidate outliers under strong selective sweeps. And the selective sweep regions were further genetically annotated by Bedtools v2.17.0 [29] to list the genes. The genes were finally subjected to GO analysis and KEGG analysis using DAVID 6.8 [30] and KOBAS 3.0 to annotate functions [31].

### Gene expression analyses

Gene expression data that were generated in a previous global transcriptome project in Jinding ducks were used for gene expression analyses (deposited in GSA: CRA005297). The data were from 60 samples representing five tissues involved in reproductive functions, i.e. the hypothalamus (n = 6 for 14-week-old, n = 6 for 19-week-old, and n = 6 for 30-week-old animals, respectively), pituitary gland (n = 6 for 14-week-old, n = 6 for 19-week-old, and n = 6 for 30-week-old animals, respectively), ovary (n = 6 for 30-week-old animals), oviduct (n = 12 for 30-week-old animals), and follicular membrane (n = 6 for 30-week-old animals). RNA was isolated from these tissues using the TRIzol (Takara, China) method according to the manufacturer’s instructions. The quantity and quality of the isolated total RNA were assessed with a Nanodrop spectrophotometer and by agarose gel electrophoresis. The cDNA library construction was performed according to the manufacturer’s protocol (Illumina, USA). The libraries were sequenced on the Illumina HiSeq 2500 platform, and 150-bp pair-end reads were generated. Analysis of mRNA transcript-level gene expression was performed according to the framework established by Pertea et al. [32]. Specifically, all paired clean transcriptome reads were mapped to the duck genome (IASCAAS_Peking Duck_PBH1.5, GCF_003850225.1) using the HISAT2 (V2.1.0) software. Then, the number of reads per gene was counted with the Htseq package and the counts per million (CPM) value of each gene was calculated.

## Results

### Phenotypic statistics and estimates of genetic parameters

The phenotypic statistics for each trait are in Table 1 and show that the coefficient of variation was greater than 10% for all traits, except for HP, and was largest for FCR, at 50%. The phenotypic values for HP, FI, DFI, and EP tended to follow a normal distribution with small skewness and kurtosis values, as their absolute values were less than 1. For the EW and FCR, they tended to be closer to a normal distribution; therefore, we tried a logarithmic transformation for these two traits for the subsequent analysis.

**Table 1:** Descriptive statistics for six egg production traits in the F_2_ population

	N	Mean	SD	Minimum	Maximum	Skewness	Kurtosis	CV (%)
HP (mm)	338	46.4	3.6	36.2	61.2	0.24	0.56	7.69
FI (g)	330	7112	1113	3247	10,525	− 0.05	0.41	15.65
EW (g)	330	2741	3889	1267	3633	− 1.17	2.21	14.18
FCR (%)	330	2.9	1.5	1.9	15.5	5.76	38.10	50.00
DFI (g)	330	195.6	32.0	199.1	292.4	− 0.09	0.24	16.34
EP	330	89.0	18.6	30.0	120.0	− 0.93	0.58	20.88

The difference in the number of individuals between traits are mainly due the death of some ducks before phenotyping

HP, hip-width (35-week old ducks); FI, feed intake (determined over 37 days); EW, egg weight (determined over 37 days); FCR, feed conversion ratio (determined over 37 days); DFI, daily feed intake; EP, egg production (between 226 and 329 days of age); CV, coefficient of variation; N, sample number for determination; SD, standard deviation

Estimates of genetic parameters for all traits are in Table 2. Estimates of the pedigree-based heritability ranged from 0.10 to 0.51 across traits (Table 2). Three of the traits, i.e. FI and DFI (both related to feeding intake) and HP (a body measurement) were highly heritable, i.e. with estimates higher than 0.4. FCR and EW were moderately heritable at 0.23 and 0.25, respectively, and EP was lowly heritable, at 0.1. FI was positively correlated with the four other traits at both the phenotypic and genetic levels, which reached significance (P < 0.05). EW was positively correlated with DFI and EP, respectively, but negatively correlated with FCR at both the phenotypic and genetic levels, which means that higher EW is associated with better FCR, which is favorable for poultry production. FCR was positively correlated with both food intake traits (FI and DFI) but negatively correlated with the egg-related traits (EW and EP), at both the phenotypic and genetic levels. Among all pairs of traits, the highest correlations were obtained between EP and EW, with a phenotypic correlation of 0.77 and a genetic correlation of 0.57.

**Table 2:** Estimates of genetic parameters for egg production traits in the F_2_ population

	HP	FI	EW	FCR	DFI	EP
HP	*0.43*	0.14*	0.04	0.01	0.11*	− 0.04
FI	0.127*	*0.51*	0.54**	0.26**	1.00**	0.33**
EW	0.04	0.53**	*0.25*	− 0.67**	0.54**	0.77**
FCR	0.05	0.19**	− 0.64**	*0.23*	0.26**	− 0.55**
DFI	0.10	0.96**	0.50**	0.19**	*0.51*	0.41**
EP	− 0.09	0.23**	0.57**	-0.40**	0.33**	*0.10*

On the diagonal, in italic characters heritability estimates

Upper triangle, phenotypic correlations

Lower triangle, genetic correlations

HP, hip-width (35-week old ducks); FI, feed intake (determined over 37 days); EW, egg weight (determined over 37 days); FCR, feed conversion ratio (determined over 37 days); DFI, daily feed intake; EP, egg production (between 226 and 329 days of age)

Significant differences among groups are indicated as **P < 0.01 and *P < 0.05

### Genome-wide association study

**Fig. 1:**
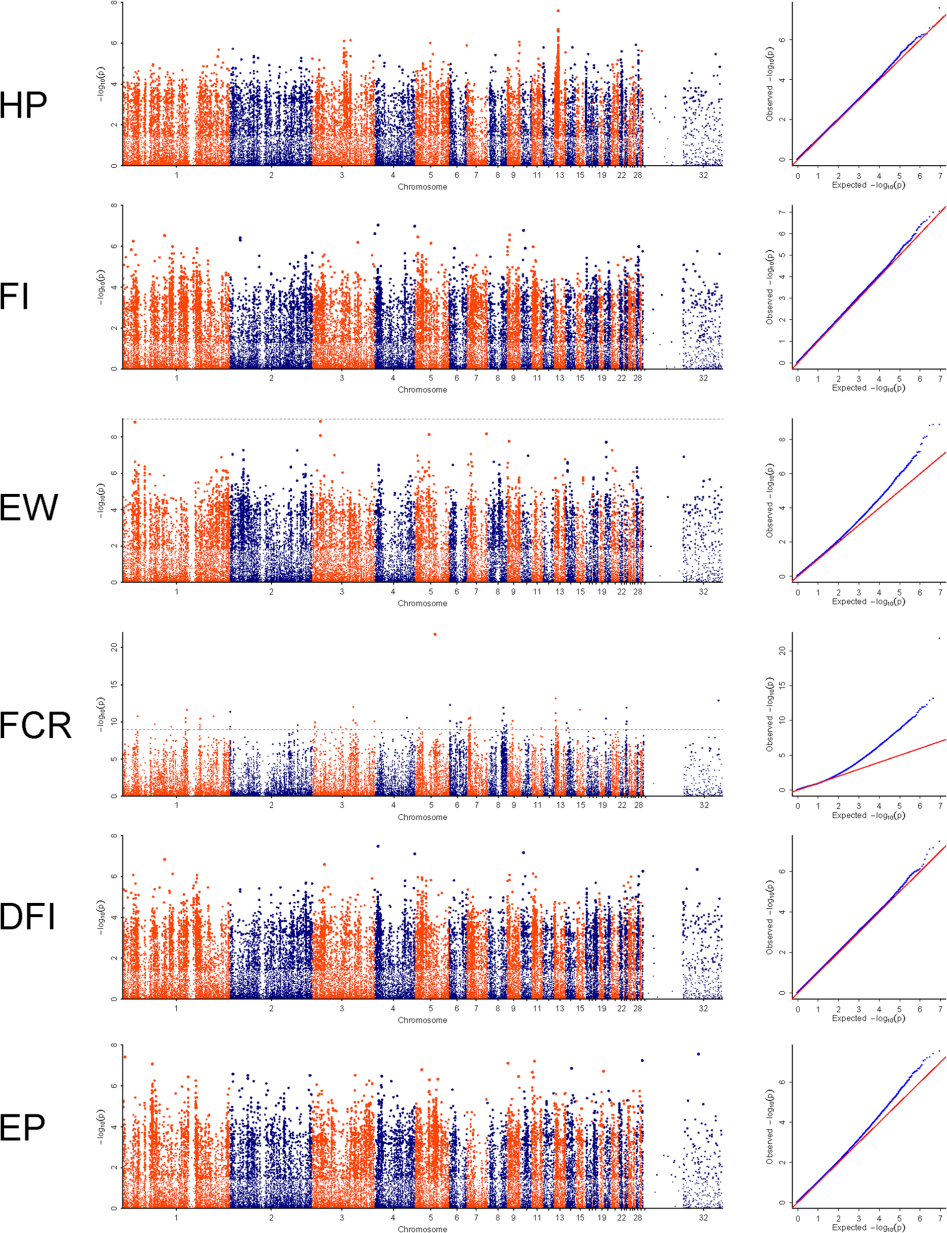
Manhattan and Q–Q plots of the genome-wide association studies for the six egg production traits analyzed. Each dot in the Manhattan plot represents an SNP in the dataset. The black dotted lines indicate the *Bonferroni* corrected significance threshold (− log_10_ *P* = 8.16). The Manhattan plots indicate the − log_10_ (*P*) values for genome-wide SNPs (y-axis) against their corresponding position on each chromosome (x-axis), while the Q–Q plots show the expected − log_10_ (*P*) vs. the observed − log_10_ (*P*). HP, hip-width; FI, feed intake (37 days); EW, egg weight (37 days); FCR, feed conversion ratio (37 days); DFI, daily feed intake; EP, egg production (between 226 and 329 days of age)

The Manhattan and quantile–quantile (Q–Q) plots are shown in Fig. 1. The *Bonferroni*-corrected threshold − log_10_(*P*) to identify significant marker-trait associations was set at 8.16. No SNPs were identified as significantly associated with HP, FI, DFI, and EP. Only four SNPs were significantly associated with EW, of which two were located on chromosome 3 (*P* = 1.38 × 10^–9^; Fig. 1), and the other two on chromosomes 1 and 7. The small number of significant SNPs associated with EW on these chromosomes could not support a reliable candidate genomic region.

The Q–Q plots for FCR revealed that SNPs deviated from the distribution under the null hypothesis, which indicated a moderate association between the SNPs and the phenotype (Fig. 1). In total, 155 SNPs passed the *Bonferroni*-corrected significance threshold (− log_10_(*P*) ≥ 8.16). These SNPs contributed to the formation of at least 11 GWAS peaks. The top three significant SNPs were on chromosome 5 at 37,345,836 bp (*P* = 1.79 × 10^–22^), on chromosome 13 at 2,196,728 bp (*P* = 7.05 × 10^–14^), and on chromosome 6 at 1,017,750 bp (*P* = 5.48 × 10^–13^). The genomic regions underlying the 11 significant GWAS peaks harbored at least 126 genes (see Additional file 4: Table S3), which were classified by gene ontology (GO) analysis; the most significant GO terms were cytoplasm, positive regulation of transcription from the RNA polymerase II promoter, and protein binding for the cellular component (CC), biology process (BP) and molecular function (MF) categories (see Additional file 5: Fig. S2 and Additional file 6: Table S4). According to the KEGG enrichment analysis, the top three significant enriched pathways were related to glycosaminoglycan biosynthesis and selenocompound metabolism. The ovarian steroidogenesis pathway was also significantly enriched, with an enrichment factor of 10.2%. Five genes, including *PLA2G4B* (chromosome 5), *IGF1R* (chromosome 11), *IGF1* (chromosome 1), *PLA2G4F* (chromosome 5), and *HSD17B7* (chromosome 8), were present in this pathway, which are also involved in reproduction functions (see Additional file 7: Fig. S3 and Additional file 8: Table S5). Although several pathways were significantly enriched and the identified candidate genes are known to play essential roles in egg production, it is difficult to provide reliable evidence to support any one of them in duck egg production based the GWAS results only.

### Candidate genomic regions for FCR based on combined analyses of GWAS and signatures of selection

The Pekin duck was domesticated from the mallard, and they are highly differentiated in egg production efficiency. In this study, we tried to reveal the genomic changes underlying egg production efficiency that occurred during the domestication of the Pekin duck from the mallard. We downloaded the whole-genome re-sequencing data of 96 ducks (62 Mallard and 34 Pekin ducks) and re-analyzed them based on the latest duck reference genome (IASCAAS_Peking Duck_PBH1.5, GCF_003850225.1) to screen for genomic regions and genes that have been under selection based on a selective sweep analysis using the *F*_ST_ and Pi indices between a mallard and Pekin ducks. We considered the windows with the top 5% values for the *F*_ST_ and log_2_ (θπ ratio) simultaneously as candidate outliers under strong selective sweeps. We detected 34 selective regions that harbored 28 genes.

Then, we combined the GWAS results with the detected signatures of selection to screen for candidate genomic regions contributing to FCR in ducks. The GWAS peaks on chromosomes 13, 3, and 6 overlapped with regions of the genome with signatures of selection (see Additional file 9: Fig. S4). This suggests that these regions harboring QTL were not only associated with FCR in the GWAS, but also were under selection during domestication. Thus, we focused on these three regions to identify candidate genes.

**Fig. 2:**
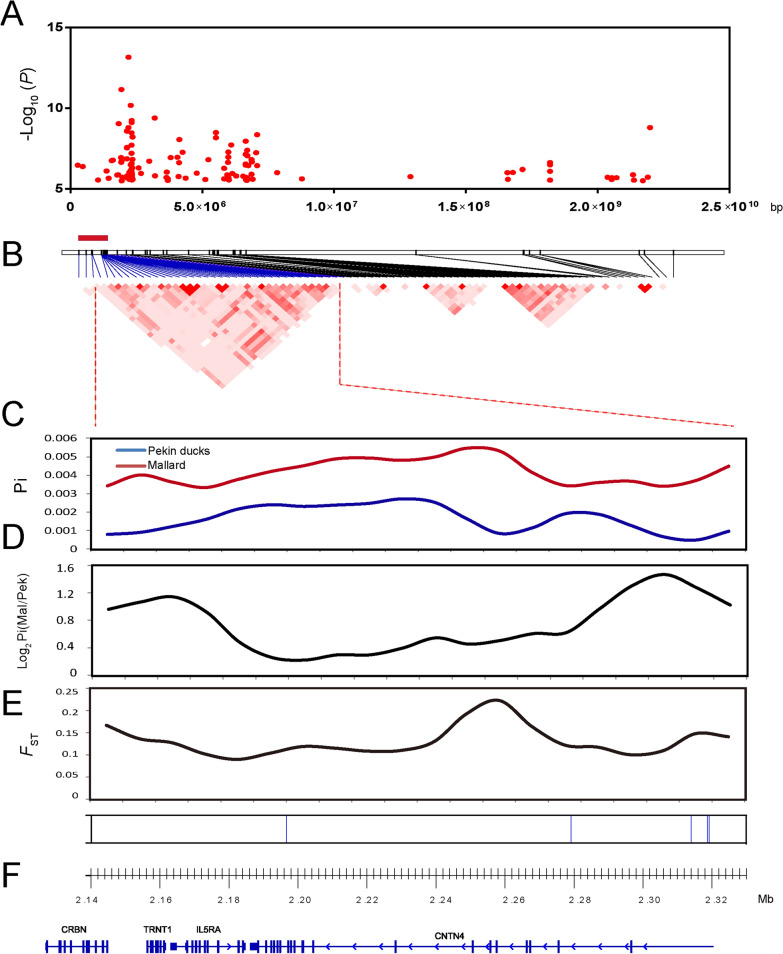
Analyses of the signatures of selection of the candidate region on chromosome 13 that affects FCR. **a** GWAS peaks located on chromosome 13. **b** Linkage disequilibrium (LD) of the significant SNPs. The red rectangle represents the region in LD with the leader SNP from the GWAS (Chr13:2,196,728, *P* = 7.05 × 10^–14^). **c** Average nucleotide diversities (Pi-value) of SNPs located in the sliding windows of the candidate region. **d** log_2_ Pi (mallard/Pekin duck) of the sliding windows. **e** Population differentiation analysis. **f** Each vertical blue line represents an SNP that reached the significance threshold of − log_10_ *P* = 8.16. The annotated genes are located in the candidate region according to the newly updated duck reference genome (IASCAAS_Peking Duck_PBH1.5, GCF_003850225.1)

**Fig. 3:**
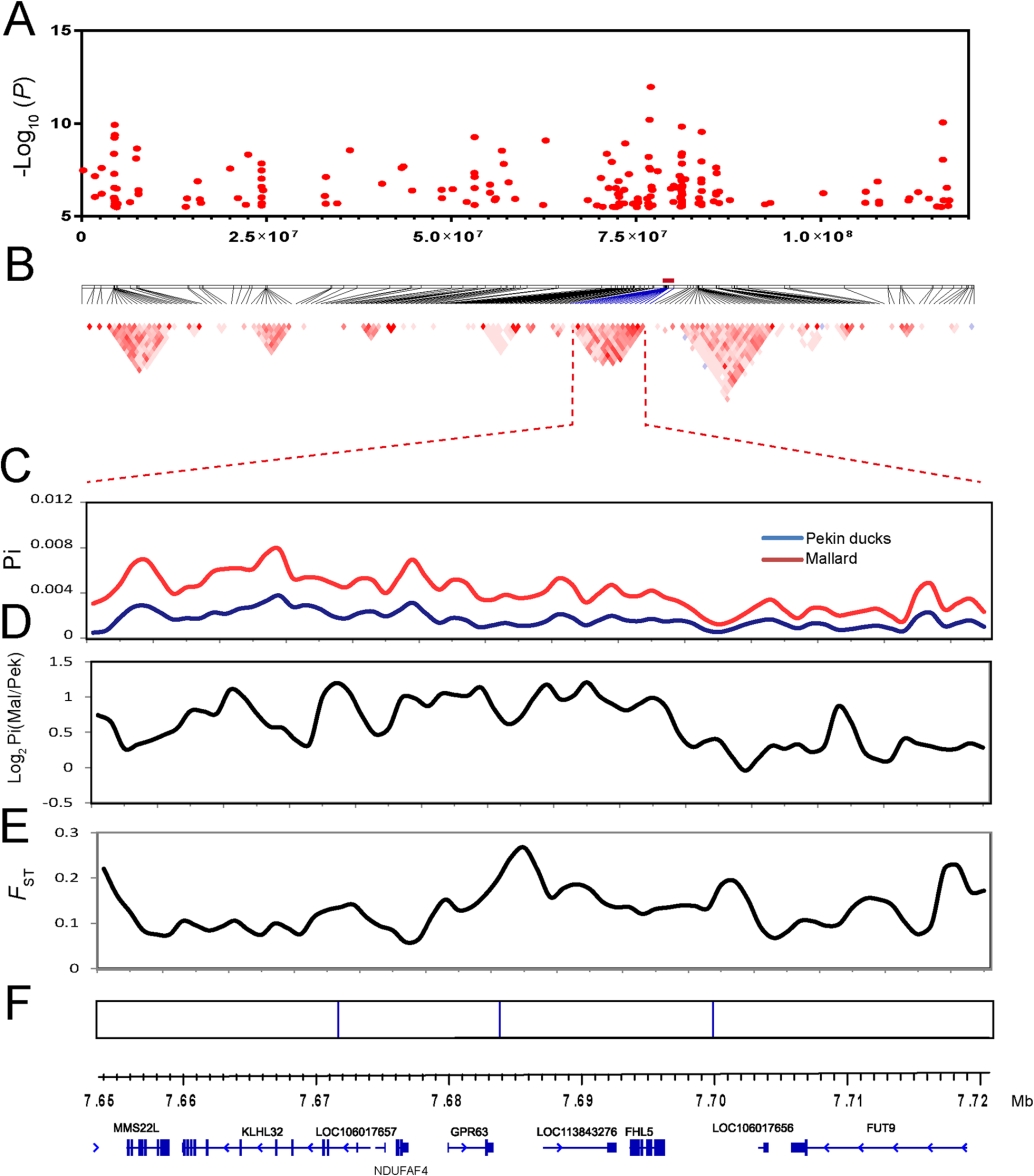
Analyses of the signatures of selection of the candidate region on chromosome 3 that affects FCR. **a** GWAS peaks located on chromosome 3. **b** Linkage disequilibrium (LD) of the significant SNPs. The red rectangle represents the region in LD with the leader SNP from the GWAS (Chr3:76,991,524, *P* = 1.06 × 10^–12^). **c** Average nucleotide diversities (Pi-value) of SNPs located in the sliding windows of the candidate region. **d** log_2_ Pi (mallard/Pekin duck) of the sliding windows. **e** Population differentiation analysis. **f** Each vertical blue line represents an SNP that reached the significance threshold of − log_10_ *P* = 8.16. The annotated genes are located in the candidate region according to the newly updated duck reference genome (IASCAAS_Peking Duck_PBH1.5, GCF_003850225.1)

**Fig. 4:**
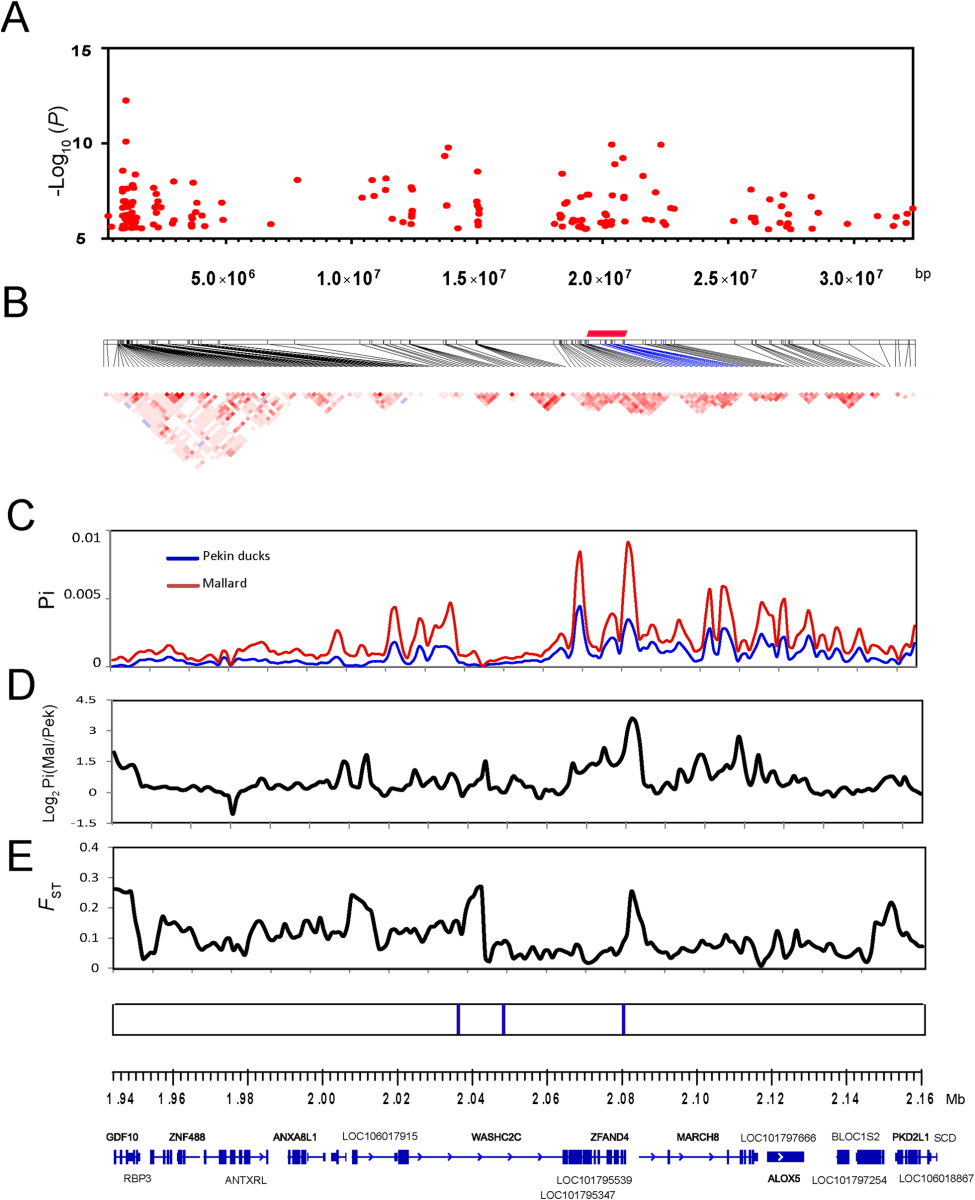
Analyses of the signatures of selection of the candidate region on chromosome 6 that affects FCR. **a** GWAS peaks located on chromosome 6. **b** Linkage disequilibrium (LD) of the significant SNPs. The red rectangle represents the region in LD with the leader SNP from the GWAS (Chr6:20,356,803, *P* = 1.14 × 10^–10^). **c** Average nucleotide diversities (Pi-value) of SNPs located in the sliding windows of the candidate region. **d** log_2_ Pi (mallard/Pekin duck) of the sliding windows. **e** Population differentiation analysis. **f** Each vertical blue line represents an SNP that reached the significance threshold of − log_10_ *P* = 8.16. The annotated genes are located in the candidate region according to the newly updated duck reference genome (IASCAAS_Peking Duck_PBH1.5, GCF_003850225.1)

To identify genomic regions for positional candidate genes, LD analysis based on the significant GWAS SNPs was performed and the haplotype blocks were visualized using the Haploview software [33]. Based on the strong LD associated with each GWAS leader SNP (Chr13:2,196,728, *P* = 7.05 × 10^–14^; Chr3:76,991,524, *P* = 1.06 × 10^–12^; Chr6:20,356,803, *P* = 1.14 × 10^–10^), the three previously identified overlapping regions, between the GWAS and selection analysis, were confined to a 0.21-Mb region on chromosome 13 between positions 2,133,672 and 2,334,566 bp (Fig. 2), a 0.69-Mb region on chromosome 3 between positions 76,530,738 and 77,219,884 bp (Fig. 3), and a 2.16-Mb region on chromosome 6 between positions 19,438,854 and 21,603,295 bp (Fig. 4). Within these candidate regions, the average Pi values of the sliding windows showed greater nucleotide diversities in mallards than in Pekin ducks. Moreover, the log_2_ Pi (mallard/Pekin duck) and *F*_ST_ also suggested that these regions were in soft selective sweeps (Figs. 2, 3 and 4). Annotated genes in these regions were identified as positional candidate genes for FCR, including five genes on chromosome 13 (*LOC106017218*, *CRBN*, *TRNT1*, *IL5RA,* and *CNTN4*) (Fig. 2), nine genes on chromosome 3 (*MMS22L*, *KLHL32*, *LOC106017657*, *NDUFAF4*, *GPR63*, *FHL5*, *LOC106017656*, *FUT9*, and *MANEA*) (Fig. 3), and 17 genes on chromosome 6 (*GDF2*, *RBP3*, *ZNF48*8, *ANTXRL*, *ANXA8L1*, *LOC106017915*, *LOC101795151*, *LOC101795347*, *LOC101795539*, *ZFAND4*, *ALOX5*, *LOC101797666*, *BLOC1S2*, *LOC101797254*, *PKD2L1*, *LOC106018867*, and *SCD*) (Fig. 4).

### Candidate genes for FCR based on transcriptome analyses

**Fig. 5:**
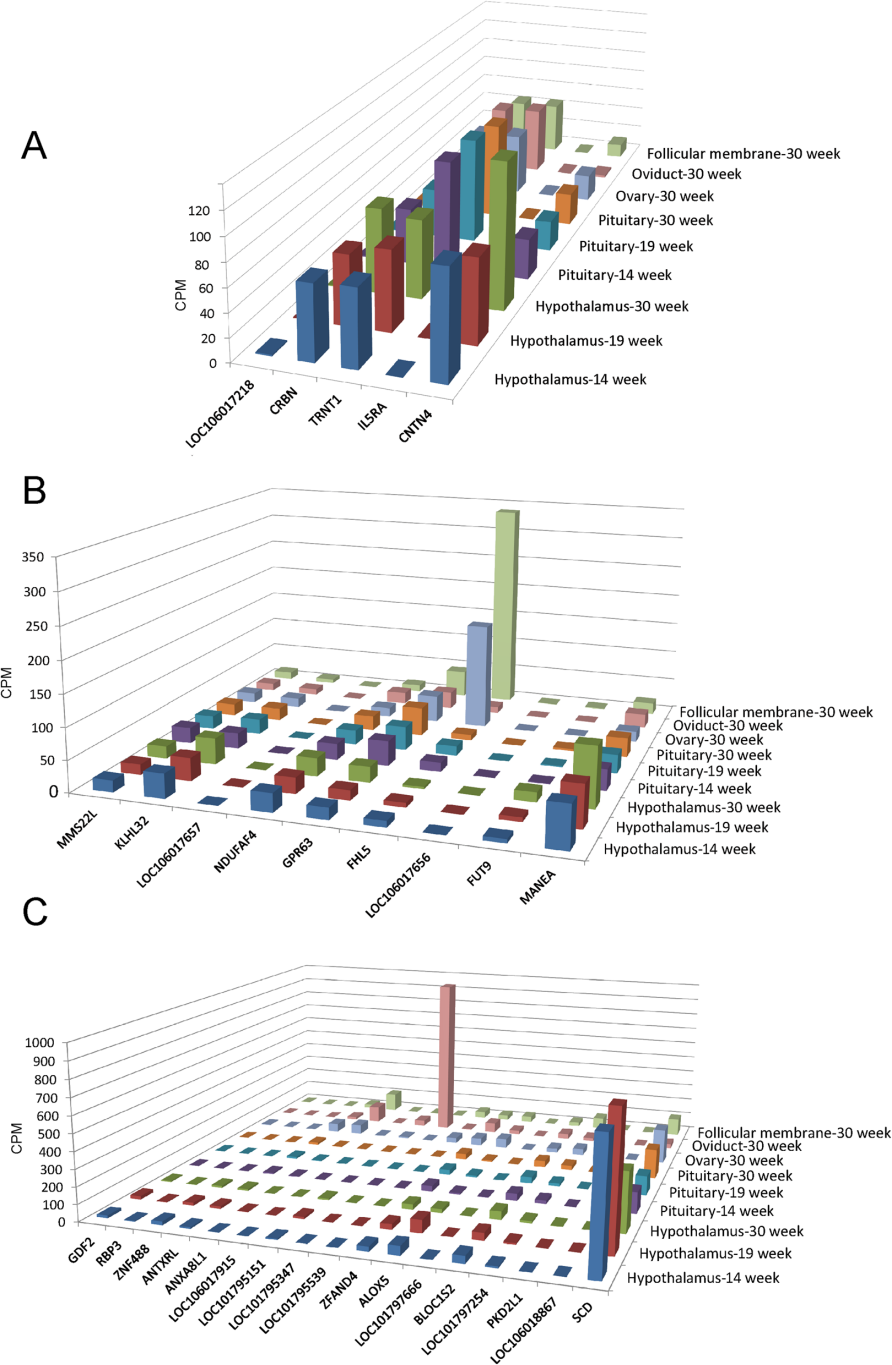
mRNA expressions of candidate genes for FCR. **a** Candidate genes located in a 0.21-Mb region on Chr13 (Chr13: 2,133,672–2,334,566 bp). **b** Candidate genes located in a 0.69-Mb region on Chr3 (Chr3: 76,530,738–77,219,884 bp). **c** Candidate genes located in a 2.16-Mb region on Chr6 (Chr6:19,438,854–21,603,295 bp). The data originate from a database of duck gene expressions determined by transcriptome analyses of 60 samples from five tissues, including the hypothalamus, pituitary gland, ovary, oviduct, and follicular membrane

For laying ducks, FCR is influenced by many direct factors, such as feed intake behavior, digestion and absorption capacity, and egg production ability [34]. Therefore, the candidate genes that participate in the regulation of any one of the above physiological and biochemical processes may ultimately affect FCR. Based on the transcriptome data (deposited in GSA: CRA005297), we checked the transcriptional levels of all the candidate genes in the hypothalamus, pituitary gland, ovary, oviduct, and follicular membrane (Fig. 5), since these organs and tissues could have a role in regulating the feed intake behavior and laying performance of ducks. Among the candidate genes on chromosome 13, *CNTN4* was highly expressed in nervous tissues (CPM > 96 in the hypothalamus, CPM > 29 in the pituitary gland), whereas *TRNT1* and *CRBR* were expressed in all investigated tissues. Among the candidate genes on chromosome 3, *MANEA* was highly expressed in all investigated tissues, with *FHL5* being the most highly expressed gene in the ovary and the follicular membrane, whereas *GPR63*, *NDUFAF4*, *KLHL32*, and *MMS22L* had relatively moderate expression levels in all investigated tissues. Among the candidate genes on chromosome 6, *SCD* a key gene that regulates lipid metabolism had a moderate expression level in all tissues, with the highest expression level in the hypothalamus. *LOC101795347* was annotated as a lncRNA and had the highest expression level in the oviduct, an essential organ for egg formation, and *ZNF488*, *ANTXRL*, *ZFAND4* and *ALOX5* were moderately expressed in the ovary, oviduct, and the follicular membrane of ducks.

**Fig. 6:**
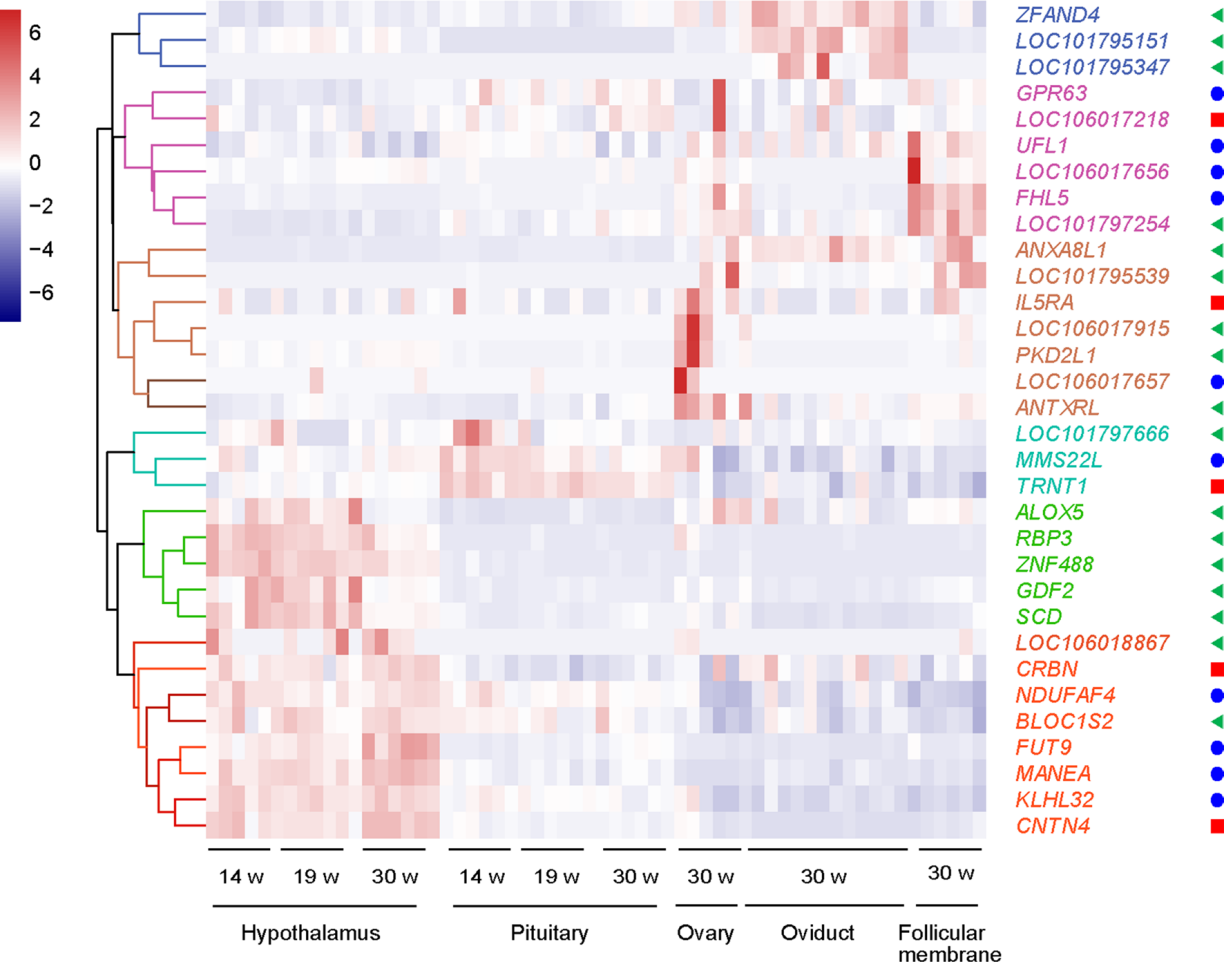
Cluster analysis of gene expression profiles of candidate genes for FCR. The red squares at the right of the gene names represent the genes located on Chr13, the green triangles represent the genes located on Chr3, and the blue circles represent the genes located on Chr6

We also checked whether there were any potential regulatory relationships among these candidate genes. Using the STRING database, analysis of protein-to-protein interactions (PPI) showed that several proteins have potential interactive relations, although there was no other strong evidence to support regulatory relationships between them (see Additional file 10: Fig. S5). Since genes that have similar functions or within regulatory relationships usually have similar expression profiles, we performed a gene expression cluster analysis to check whether there was a potential regulatory relationship among these candidate genes. According to the heat map (Fig. 6), all the candidate genes could be classified mainly into two categories—genes that are highly expressed in the ovary, oviduct, and follicular membrane, and genes that are highly expressed in nervous tissues, including the hypothalamus and pituitary gland.

## Discussion

Availability of accurate and reliable genetic parameters for quantitative traits are of great significance for designing breeding programs, predicting response to selection, and explaining genetic effects [35]. In poultry production, feed represents over 60% of the production costs. Many studies have been carried out on traits related to feed efficiency. In egg-type poultry, several heritability estimates have been reported for FCR. In this study, we found that the heritability estimates were higher than 0.4 for two feed intake-related traits (FI and DFI) and equal to 0.23 for FCR. Similar to our results, Zeng et al. [36] reported heritability estimates for FCR, FI, and residual feed intake (RFI) of 0.19, 0.22, and 0.27 in Shaoxing ducks, and of 0.19, 0.24, and 0.24 in Jinyun ducks, respectively. These studies provide evidence that direct selection can improve the feed efficiency of laying ducks. Nevertheless, genetic parameters for feed efficiency traits in egg-type poultry of different genetic backgrounds are still lacking. Our findings help to decipher the genetic background of feed efficiency and egg production traits and contribute to duck breeding and genomic studies.

Our results show that the analyzed egg production traits were highly correlated with each other, both phenotypically and genetically, which indicates that they may share similar genetic components or be influenced by some pleiotropic genomic regions [37, 38]. However, based on the GWAS results, significant SNPs were detected for FCR only. Currently, 600 QTL associated with egg production traits have been identified in chickens according to the records of the chicken QTL database [14]. Most of the GWAS have been performed based on SNP arrays. Currently, the commercial high-density chip that is widely used in chickens is a 600 k SNP array [39]. In the past few years, the reduction in the cost of whole-genome sequencing technologies has favored its widespread application and more and more GWAS now use whole-genome SNPs [40–42]. In our GWAS, we used more than 7 million SNPs in the duck genome to identify significant marker-trait associations with a strict *Bonferroni*-corrected threshold (− log_10_(*P*) ≥ 8.16). Because of the multiple statistical tests and the large number of SNPs, it was more difficult to identify significant associations [43], and in addition, the limited sample size used may have affected our findings. Thus, SNPs with small effects may be missed because they do not reach the statistical threshold.

FCR is a very complex trait because it is influenced by feed intake behavior, digestion and absorption capacity, and egg production ability [34]. Pekin ducks have become a world-famous farm breed with high performances in meat and egg yields. We assume that FCR of Pekin ducks and mallards differ largely because of the long domestication process of the former from the latter and of closed breeding populations. In this study, the coefficient of variation of phenotypic values for FCR was 50% in the F_2_ population that was constructed using Pekin ducks and mallards, which suggests that the phenotypic value of FCR was different in the F_2_ population.

Through GWAS, we identified 18 genomic regions that were associated with FCR-related traits but these association signals were not strong, as shown by the Q–Q results (Fig. 1), which displayed moderate leftward deflections of the observed distribution. This phenomenon is often attributed to “spurious inflation” and would be expected under a polygenic architecture. In a recent review for complex traits, Yang et al. [44] assumes that the genetic variations that are below the GWAS threshold could also contribute largely to heritability and pointed out the polygenic characteristics of complex traits.

We identified 126 annotated genes in the 18 significant genomic regions. Based on GO and KEGG enrichment analyses, genes with the GO terms of glycosaminoglycan biosynthesis and selenocompound metabolism were found to be enriched in the significant regions. These terms are related to hormone synthesis and metabolism in reproduction [45, 46]. In addition, some of the positional candidate genes are involved in ovarian steroidogenesis, including *PLA2G4B*, *IGF1R*, *IGF1*, *PLA2G4F*, and *HSD17B7*. These findings suggest that the identified genomic regions may contain the actual causative loci that affect FCR.

An important research strategy to reveal the genetic basis of complex traits is to combine signatures of selection and GWAS results [47, 48], especially for traits that are assumed to have been strongly selected for during domestication. Historically, farmers probably selected poultry for egg production, which is likely to have affected egg weight and egg laying rate, and thus the effect on FCR may have been a correlated response to selection on egg production. By integrating GWAS and analyses of signatures of selection, we identified three genomic regions related to FCR on chromosomes 13, 3, and 6, respectively. In the end, 31 of the 126 positional candidate genes related to FCR were supported by LD analysis and in which the GWAS leader SNP was located (Chr13:2,196,728, *P* = 7.05 × 10^–14^; Chr3:76,991,524, *P* = 1.06 × 10^–12^; Chr6:20,356,803, *P* = 1.14 × 10^–10^). Some of these functional candidate genes were highly expressed in tissues with reproductive functions. *FHL5* may be involved in the regulation of spermatogenesis [49], and among the tissues investigated here, its expression was highest in the ovary and the follicular membrane. Some of the functional candidate genes are involved in essential functions of metabolic processes, e.g., *TRNT1* is expressed in all investigated tissues, and has basic functions in the mRNA translation process to produce peptides [50]; *NDUFAF4* is involved in energy metabolism in the mitochondria [51]; and *SCD* is a crucial gene that regulates lipid metabolism [52]. We also found that *LOC101795347*, as a lncRNA, had the highest expression in the oviduct, which is an essential organ for egg formation. Some of the functional candidate genes were highly expressed in nervous tissues (hypothalamus and pituitary gland) and it is well known that genes associated with neural processes were initially under selection during the domestication of animals [53–56]. Some of the genes that are highly expressed in nervous tissues are involved in feed intake behaviors or linked to stress responses, potentially giving a calmer, less stressed bird that may be more feed-efficient than the wild mallard duck, i.e., they could affect FCR (reducing FI and increasing EW). Examples of such genes include *CNTN4,* which has a role in synaptogenesis [57], *CRBN,* which is related to the intelligence quotient of humans [58], and *GPR63,* which has a function in the brain [59].

The integration of GWAS and analyses of signatures of selection helped us identify three genomic regions that affect FCR in the duck, with positional candidate genes that are involved in nervous activity, metabolism, and reproduction processes. However, this does not mean that the other regions identified by GWAS do not contribute to the genetic architecture underlying FCR. Detection of additional genomic regions that are involved in FCR and egg production traits requires further studies, by, e.g., increasing sample size or performing functional analyses. Nevertheless, our findings not only provide GWAS results that shed light on the genetics of duck feed efficiency but also deepens our understanding of the genomic changes underlying the domestication process. By incorporating the identified SNPs into breeding programs, selection for FCR in ducks can be implemented to save production costs.

## Conclusions

We have reported genetic parameters for six egg production traits in ducks based on an F_2_ population that was constructed from mallard and Pekin ducks. We performed a GWAS to identify genetic loci that underlie these six egg production traits but only detected 11 genomic regions, which were all associated with FCR. Three of these regions, on chromosomes 3, 6, and 13, were confirmed by analyses of signatures of selection. These three genomic regions harbor 31 functional candidate genes, among which, *CNTN4*, *CRBR*, *GPR63*, *KLHL32*, *FHL5*, *TRNT1 MANEA*, *NDUFAF4*, and *SCD* are the main candidate genes, based on their high expression in nervous and reproductive tissues. Our study is the first to provide genomic regions associated with FCR in ducks, which will be useful for genomic selection in duck breeding. Our data also illustrate that the genes that appear to have been selected in ducks during their domestication are related to nervous, reproduction and metabolic functions.

## Supplementary Information

**Additional file 1: Table S1.** Composition of the diet (g/kg as fed) used to feed the ducks during the laying period.

**Additional file 2: Figure S1.** Photo of the individual cages (1 m by 1 m) used for each duck.

**Additional file 3: Table S2.** Clean read data for 62 mallard and 34 Pekin ducks obtained by whole-genome re-sequencing and downloaded from NCBI.

**Additional file 4: Table S3.** Annotation of the genes detected with the top 2000 SNPs associated with FCR in the GWAS. The top 2000 SNPs were screened according to − log_10_ (*P*). The cells highlighted in red on the chromosome line represent SNPs that reached the *Bonferroni* threshold. The average *F*_ST_ and Pi of the slide windows are also provided based on the analysis of selective sweeps.

**Additional file 5: Figure S2.** The top five GO terms by enrichment factor with a significant *P* value, which were enriched in the categories cellular component (CC), biology process (BP), and molecular function (MF), respectively. The genes used for GO analysis were from the annotated genes according to the top 2000 SNPs in the GWAS.

**Additional file 6: Table S4.** GO enrichment results of the genes annotated based on the top 2000 SNPs in GWAS.

**Additional file 7: Figure S3.** The top 15 KEGG pathways by enrichment factor with significant P values. The genes used for KEGG analysis were from the annotated genes according to the top 2000 SNPs in GWAS.

**Additional file 8: Table S5.** The KEGG enrichment results of genes annotated by the top 2000 SNPs in GWAS.

**Additional file 9: Figure S4.** Genome-wide association and selective sweep analyses on the candidate regions associated with FCR. After checking the GWAS association peaks on chromosomes13, 3, and 6, one by one, we observed that they overlapped with the selection target genomic supported by the sliding windows. Each blue and red dot in the image of the selection analysis represents a sliding window, while each purple dot represents an SNP site in the GWAS analysis.

**Additional file 10: Figure S5.** Protein–protein interactions (PPI) show the potential relationships between the 31 candidate genes according to the STRING database.

## Data Availability

The sequence data have been stored in Genome Sequence Archive (GSA) under the Project name of PRJCA003535 and CRA005297.
