# Supplementary material for: Genome-wide association and selective sweep analyses reveal genetic loci for FCR of egg production traits in ducks
Source: Genet Sel Evol. 2021 Dec 20;53:98. doi: 10.1186/s12711-021-00684-5 (PMC8690979; doi:10.1186/s12711-021-00684-5)
Supplement: Supplementary file 1 — Additional file 1: Table S1. Composition of the diet (g/kg as fed) used to feed the ducks during the laying period. [file 12711_2021_684_MOESM1_ESM.docx]

**Table S1.** Composition of diet (g/kg as-fed) for ducks at laying period stages

| Item | | g/kg | |
| --- | --- | --- | --- |
| Ingredient | |  | |
| Corn | | 560.0 | |
| Soybean | | 238.0 | |
| Corn gluten meal | | 100.0 | |
| Limestone | | 70.0 | |
| Dicalcium phosphate | | 15.0 | |
| Vitamin and trace mineral premix ^a^ | | 10.0 | |
| Sodium chloride | | 3.0 | |
| DL-Methionine | | 1.0 | |
| L-Lysine·HCl | | 3.0 | |
| Calculated composition | |  | |
| Metabolizable energy ^b^, MJ/kg | | 11.48 | |
| Crude protein | | 192.9 | |
| Calcium | | 30.7 | |
| Nonphytate phosphorus | | 3.5 | |
| Lysine | | 10.4 | |
| Methionine | | 4.6 | |
| Methionine + cysteine | | 7.7 | |
| Threonine | | 7.6 | |
| Tryptophan | | 2.0 | |
| Arginine | | 10.1 | |

^a^ Supplied per kilogram of total diet: Cu (CuSO_4_•5H_2_O), 10 mg; Fe (FeSO_4_•7H_2_O), 60 mg; Zn (ZnO), 60 mg; Mn (MnSO_4_•H_2_O), 80 mg; Se (NaSeO_3_), 0.3 mg; I (KI), 0.2 mg; choline chloride, 1000 mg; vitamin A (retinyl acetate), 10000 IU; vitamin D_3_ (Cholcalciferol), 3000 IU; vitamin E (DL-α-tocopheryl acetate), 20 IU; vitamin K_3_ (menadione sodium bisulfate), 2 mg; riboflavin, 15mg; thiamin (thiamin mononitrate), 2 mg; pyridoxine hydrochloride, 4 mg; cobalamin, 0.02 mg; calcium-D-pantothenate, 20 mg; nicotinic acid, 50 mg; folic acid, 1 mg; biotin, 0.2 mg.

^b^ The values are calculated according to the AME of ducks (Ministry of Agriculture of China, 2012).
