# Supplementary figures and images for: Genome-wide association and selective sweep analyses reveal genetic loci for FCR of egg production traits in ducks
Source: Genet Sel Evol. 2021 Dec 20;53:98. doi: 10.1186/s12711-021-00684-5 (PMC8690979; doi:10.1186/s12711-021-00684-5)

**
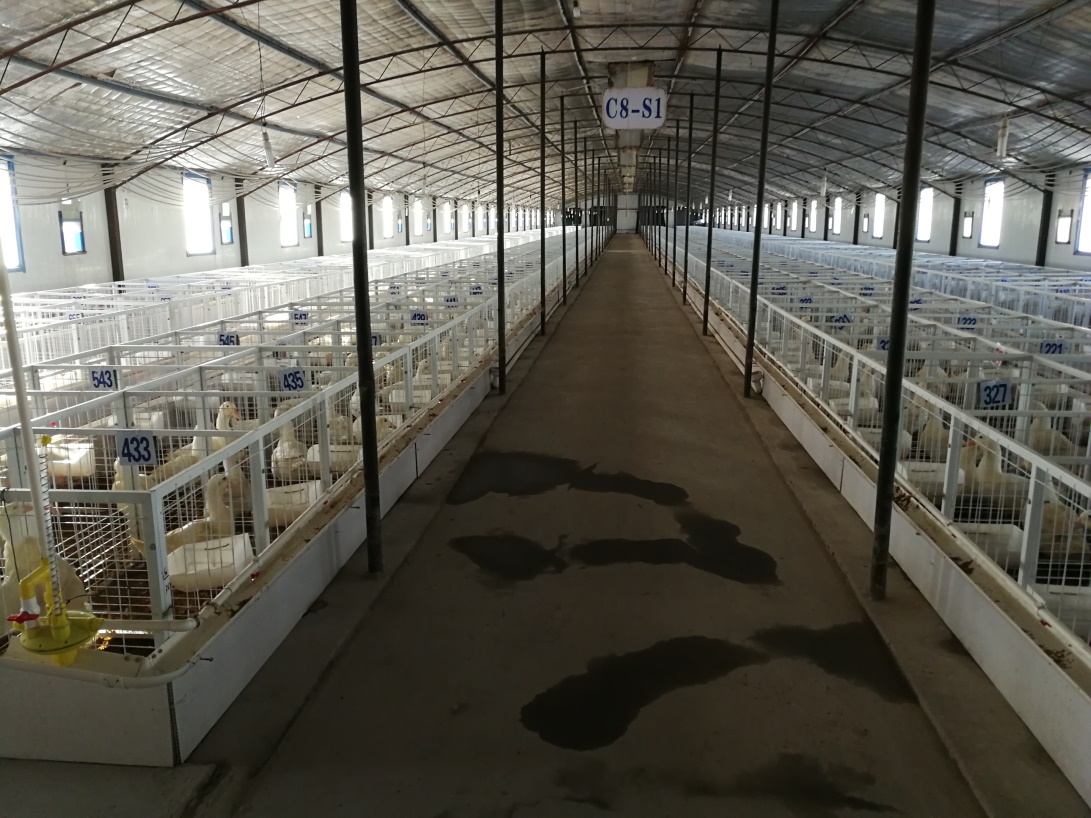
**

**Fig.S1** The individual cage used for duck. The area of cage is set up with 1m by 1m.

Supplement: Supplementary file 2 — Additional file 2: Figure S1. Photo of the individual cages (1 m by 1 m) used for each duck. [file 12711_2021_684_MOESM2_ESM.docx]

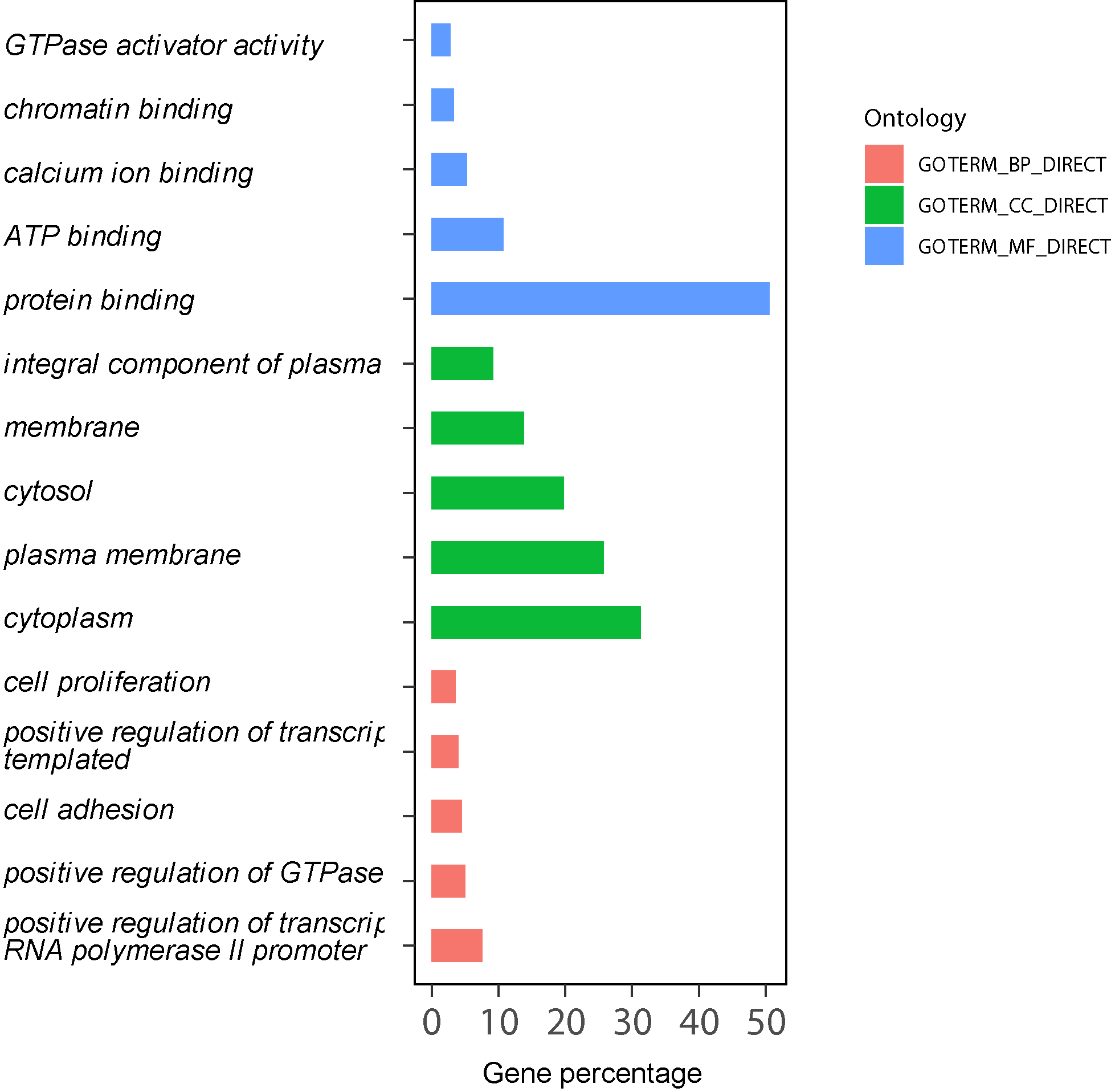

Supplement: Supplementary file 5 — Additional file 5: Figure S2. The top five GO terms by enrichment factor with a significant P value, which were enriched in the categories cellular component (CC), biology process (BP), and molecular function (MF), respectively. The genes used for GO analysis were from the annotated genes according to the top 2000 SNPs in the GWAS. [file 12711_2021_684_MOESM5_ESM.tif]

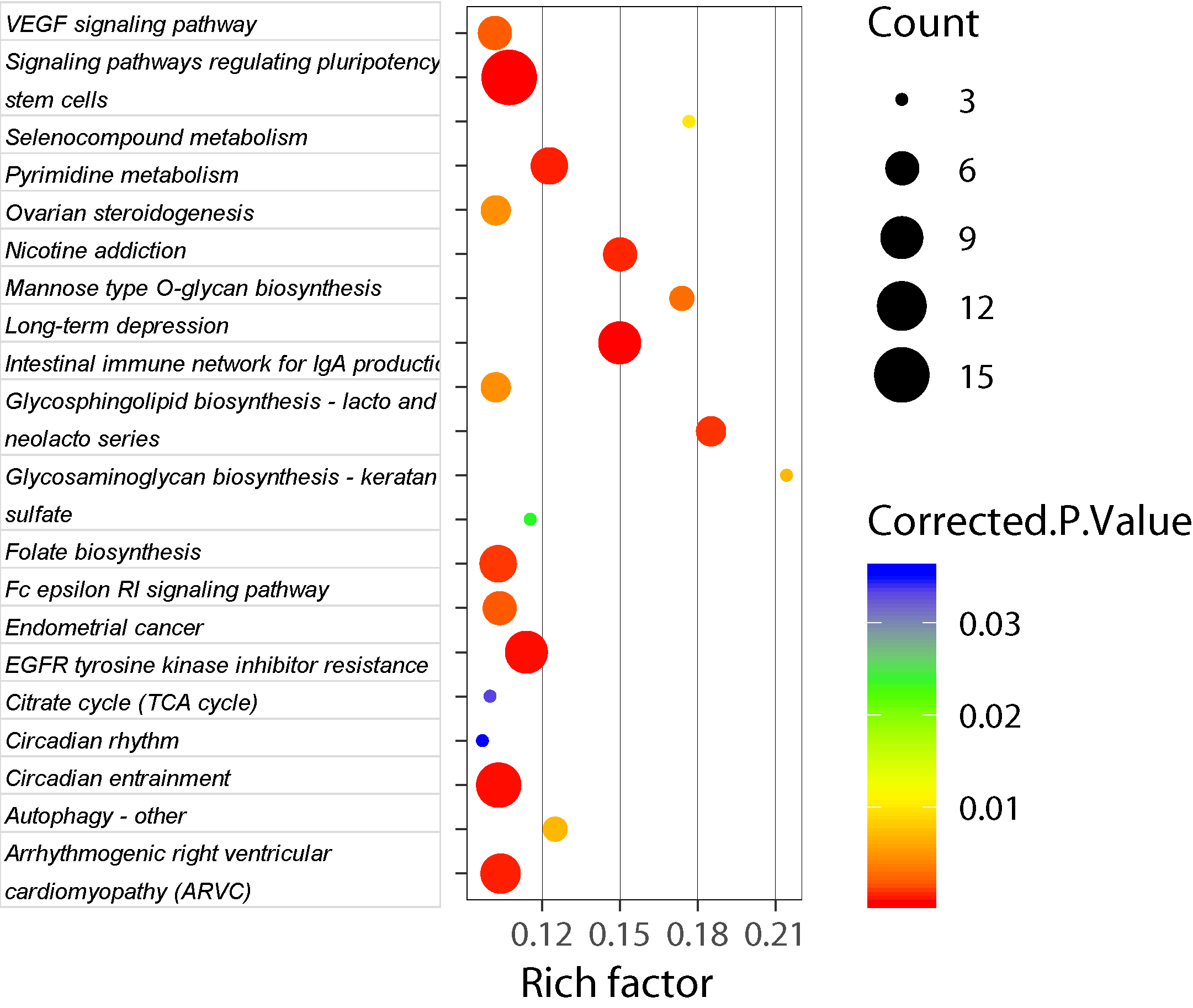

Supplement: Supplementary file 7 — Additional file 7: Figure S3. The top 15 KEGG pathways by enrichment factor with significant P values. The genes used for KEGG analysis were from the annotated genes according to the top 2000 SNPs in GWAS. [file 12711_2021_684_MOESM7_ESM.tif]

Chr1

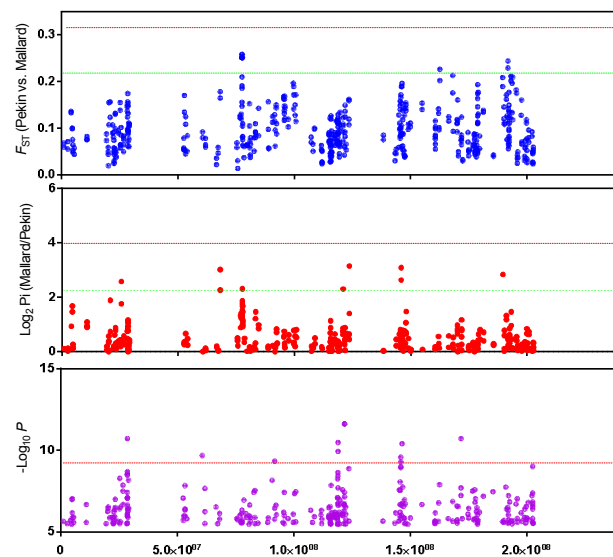

Chr2

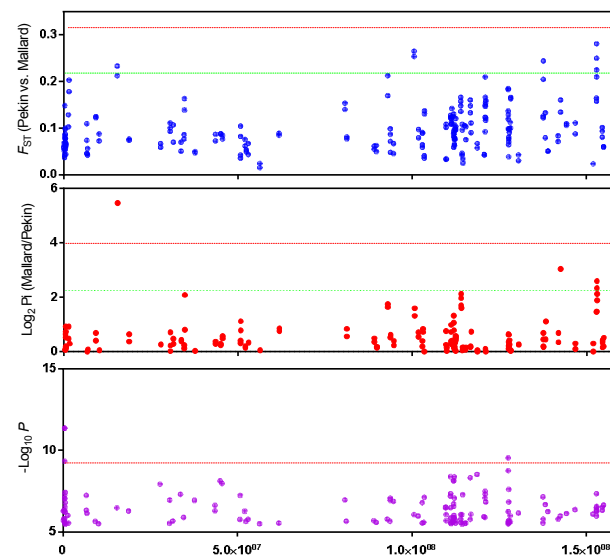

Chr4

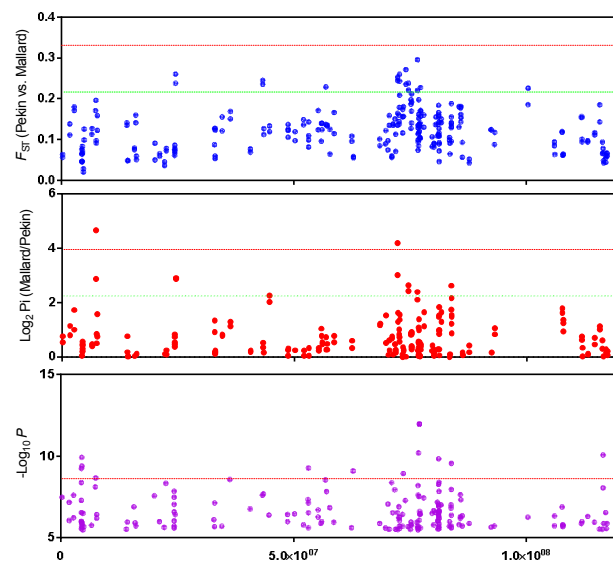

Chr5

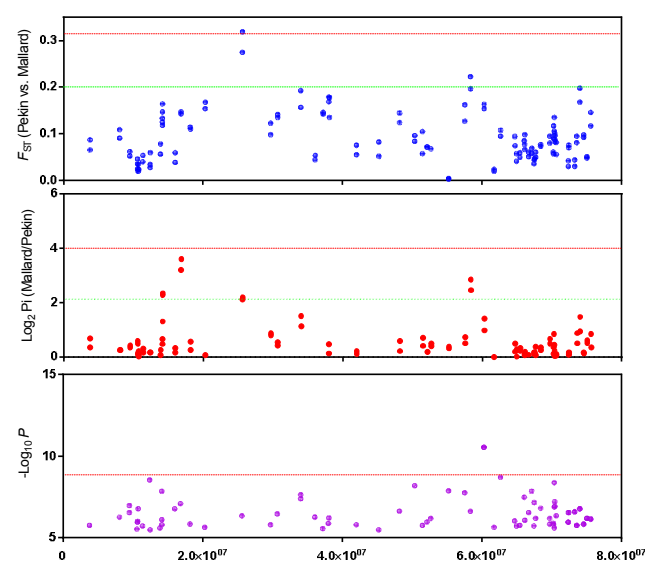

Chr7

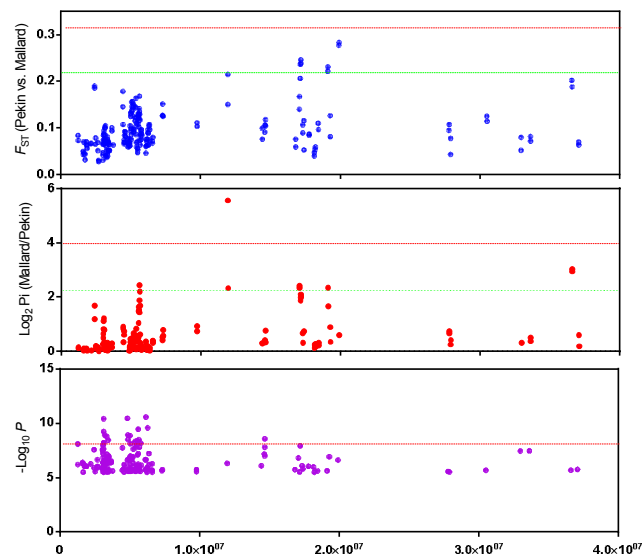

Chr8

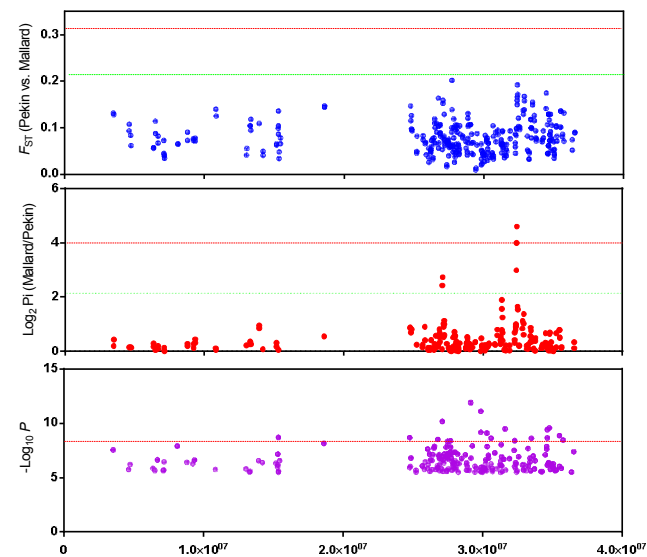

Chr9

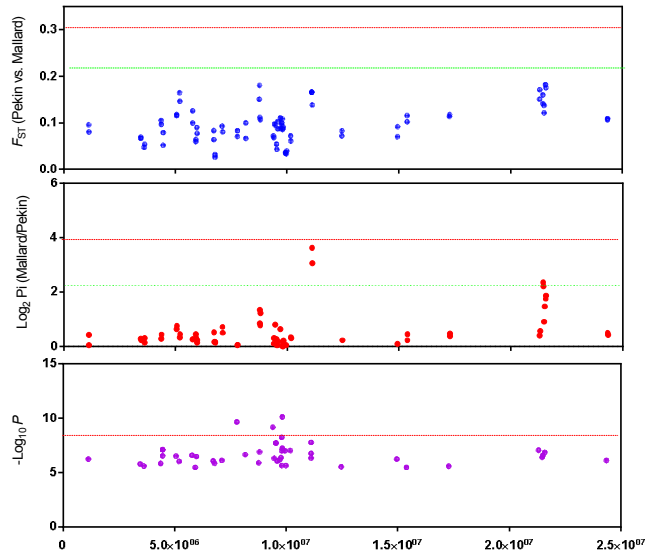

Chr24

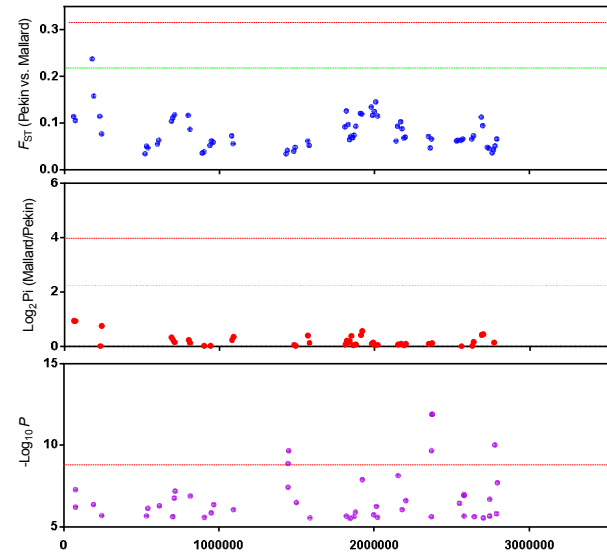

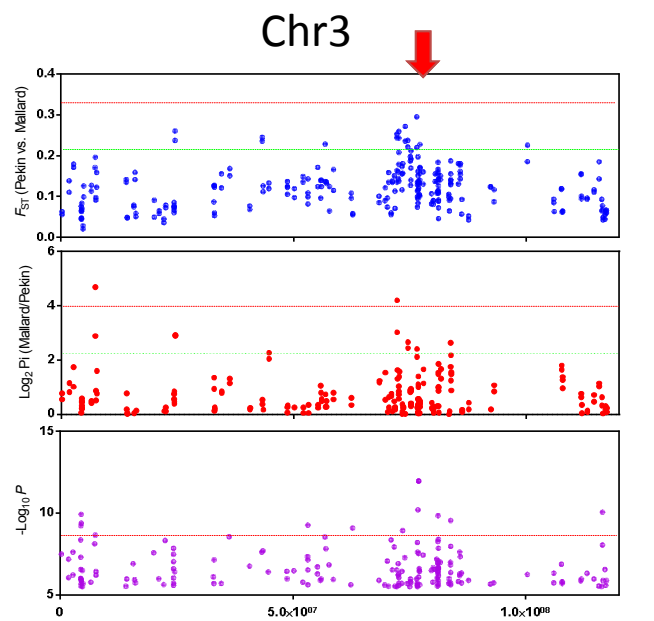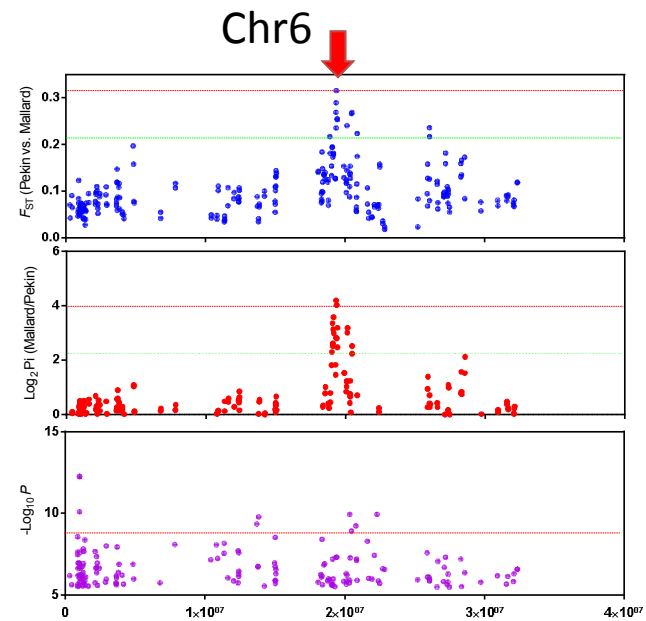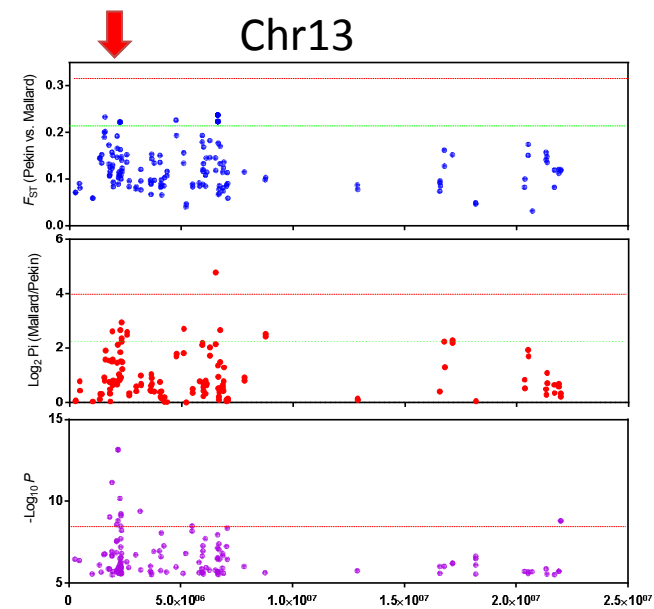

Supplement: Supplementary file 9 — Additional file 9: Figure S4. Genome-wide association and selective sweep analyses on the candidate regions associated with FCR. After checking the GWAS association peaks on chromosomes13, 3, and 6, one by one, we observed that they overlapped with the selection target genomic supported by the sliding windows. Each blue and red dot in the image of the selection analysis represents a sliding window, while each purple dot represents an SNP site in the GWAS analysis. [file 12711_2021_684_MOESM9_ESM.pdf]

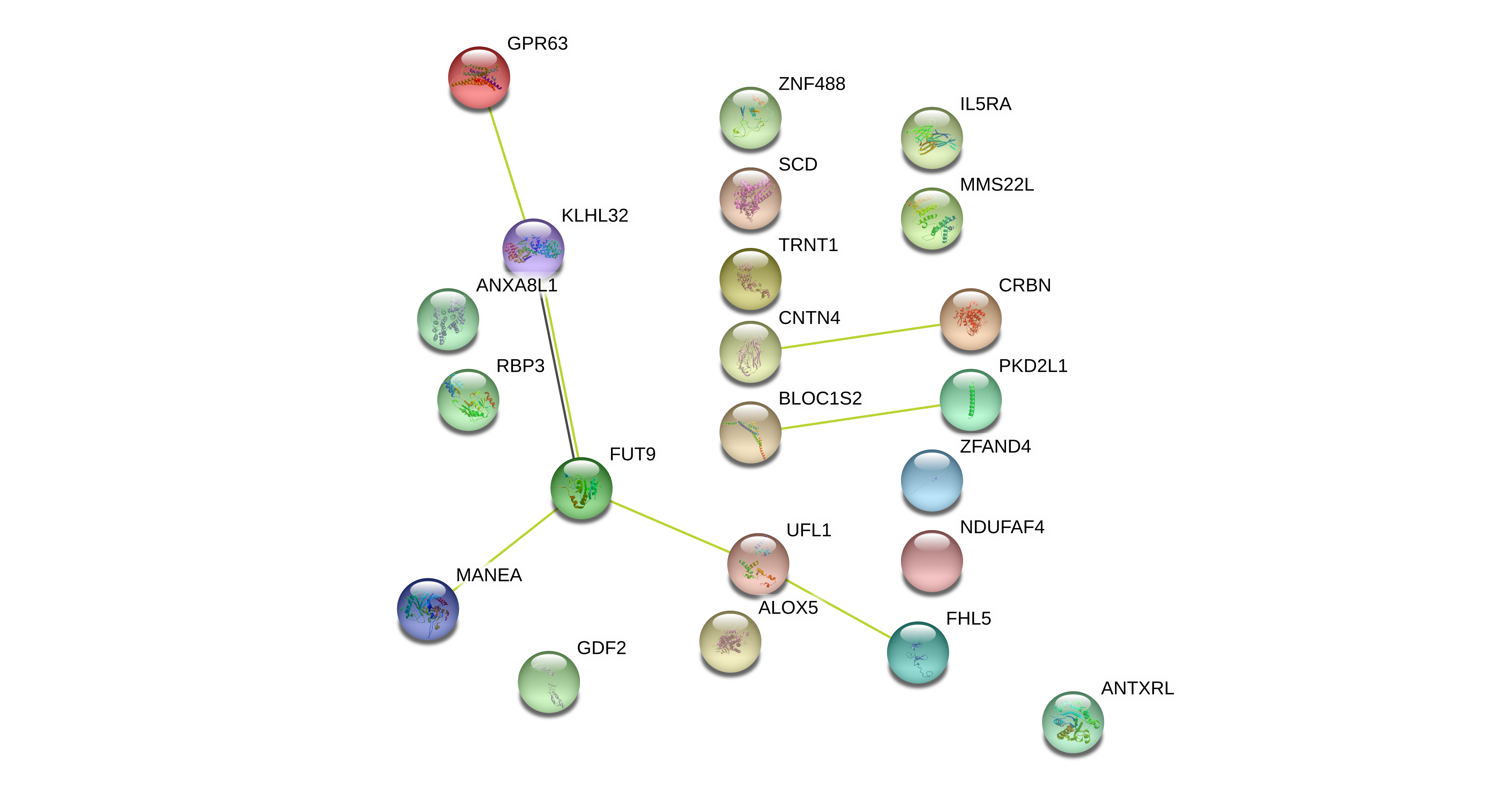

Supplement: Supplementary file 10 — Additional file 10: Figure S5. Protein–protein interactions (PPI) show the potential relationships between the 31 candidate genes according to the STRING database. [file 12711_2021_684_MOESM10_ESM.png]
